# Sox8: a multifaceted transcription factor in development and disease

**DOI:** 10.1242/bio.061840

**Published:** 2025-02-12

**Authors:** María Nazareth González Alvarado, Jessica Aprato

**Affiliations:** ^1^ Institute of Molecular Biotechnology of the Austrian Academy of Sciences (IMBA), Vienna Biocenter (VBC), 1030, Vienna, Austria; ^2^ Institut für Biochemie, Emil-Fischer-Zentrum, Friedrich-Alexander-Universität (FAU) Erlangen-Nürnberg, 91054 Erlangen, Germany

**Keywords:** Sox8, Transcription factor, Development, Nervous system, Sex determination, Disease

## Abstract

Sox8 is a transcription factor that belongs to the Sox family of high-mobility-group domain containing proteins and is closely related to Sox9 and Sox10. During prenatal development, Sox8 is expressed in several ectoderm-, endoderm- and mesoderm-derived tissues and has been implicated in processes of organogenesis and differentiation. Sox8 expression is found in several important cells such as Sertoli cells in the male gonad, glial cells, satellite cells, and chondrocytes. However, Sox8 is not essential for the proper development of any of the involved systems, as it functions redundantly with Sox9 or Sox10 and no major developmental disturbances have been noticed in its absence. Despite its perceived limited importance as a developmental regulator, Sox8 exhibits a more significant role in late development and adult tissues. Several studies highlight the importance of Sox8 for the homeostasis of adipose tissue, Sertoli cells and the blood-testis-barrier functioning, and the maintenance of myelin in the central nervous system. Emerging evidence points to *SOX8* as a promising candidate for a disease-causing gene in humans and suggests that changes in SOX8 function or expression could contribute to pathological states. For instance, genetic variants of *SOX8* have been linked to multiple sclerosis and familial essential tremor, while *SOX8* alterations have been related to poor cancer prognosis and infertility. This Review provides an overview of Sox8's versatile role in development and adult tissues as well as its lesser-known contributions to various diseases, and its potential as a therapeutic target.

## Teaser

**Summary:** Sox8 is a transcription factor involved in processes of organogenesis and differentiation during development. Recent studies suggest its involvement in diseases like Multiple Sclerosis, cancer and infertility, underscoring its potential as a therapeutic target.

## Introduction

The SRY-related HMG box (Sox) family of transcription factors play vital roles during development, regulating fundamental biological processes such as pluripotency maintenance, cell fate determination, and terminal cell differentiation. These transcription factors (TFs) are characterized by the presence of a high mobility group (HMG) domain, which was first discovered in the mammalian sex-determining gene *Sry* (Koopman et al., 1991). The HMG is a DNA-binding domain highly conserved across different species and based on the level of similarity in this sequence, Sox proteins are classified into groups A to H. In vertebrates, the SoxE group consists of Sox8, Sox9, and Sox10, which are particularly crucial during embryonic development. SoxE TFs share a high degree of sequence homology and a certain level of functional redundancy. For instance, all three SoxE factors are necessary for proper neural crest development and neuronal and glial cell differentiation (Weider and Wegner, 2017). Mutations in the *SoxE* genes often result in severe phenotypes closely related to their molecular functions. For example, mutations in the *SOX9* gene lead to Campomelic dysplasia, a severe skeletal malformation syndrome, while mutations in *SOX10* are associated with Waardenburg–Hirschsprung syndrome. To get a more in-depth understanding of SoxE TFs we recommend consulting previous reviews on the topic, such as Kamachi and Kondoh, 2013 and Weider and Wegner, 2017.

Sox8 is thought to act redundantly with Sox9 and Sox10 and to be a minor regulator of developmental processes. Consequently, Sox8 has been poorly investigated and primarily studied in conjunction with one of its relatives Sox9 or Sox10. Therefore, the specific role of Sox8 remains poorly understood.

However, emerging evidence highlights the importance of Sox8 in late development and adult tissue homeostasis. More recently, mutations in the *SOX8* gene have been linked to various human diseases. Most of the evidence in humans comes from genetic studies that link single nucleotide polymorphisms (SNPs), copy number variations (CNVs), missense mutations, or chromosomal rearrangements to infertility, and central nervous system (CNS) disorders, among other conditions. Some *in vitro* studies have reported altered protein function, and overexpression of SOX8 has been linked to negative outcomes in cancer. Yet the exact contribution of SOX8 is not fully understood, and it varies among different pathologies emphasizing the need for a deeper understanding of its unique functions.

This Review focuses on the least characterized member of the SoxE family, Sox8. It comprehensively explores Sox8's molecular structure, expression patterns, and functions in both developmental and adult tissues, with particular emphasis on its roles in the nervous system and sex determination. Additionally, it discusses Sox8's implications in various pathological processes, such as cancer, infertility, and other diseases, as well as its potential as a therapeutic target.

### Phylogenesis and molecular characteristics of Sox8

In the 1990s, the first member of the Sox family, Sry, was identified. However, the existence of these essential TFs predates the origin of metazoans (King et al., 2008). Phylogenetic analysis suggests that *SoxE* genes independently duplicated from a common ancestor in the vertebrate groups of agnathans (primitive jawless fishes) and gnathostomes (jawed vertebrates) (Bowles et al., 2000). While a *Sox9* distant-related gene is present in agnathans, *Sox8* and *Sox10* homologues are primarily found in gnathostomes (Guth and Wegner, 2008). Homologues of *Sox8* have been identified across various orders of fish, showing varying degrees of sequence similarity, expression patterns, and function. SoxE TFs exhibit a high level of conservation, not only in their genomic sequences but also in their functions. They are expressed in the gonads and ovaries of diverse phylogenetic orders and play a role in the nervous system development across different species (Ji et al., 2024; Juarez et al., 2013; Liu et al., 2012; Xia et al., 2010, 2011). Such evolutionary conservation emphasizes the intrinsic importance of SoxE TFs and how fundamental it is to elucidate their role in physiological and pathological states.

*SoxE* genes consist of three coding exons that are separated by two introns. Schepers and colleagues characterized the molecular features and chromosomal position of the TF Sox8, demonstrating that the *Sox8* gene is located within the t-complex of mouse chromosome 17. In humans, *SOX8* maps to the chromosome 16p13.3 region, where mutations are found in patients with the genetic diseases microphthalmia-cataract syndrome and alpha-thalassemia mental retardation syndrome (ATR-16) (Schepers, 2000; Schepers and Koopman, 2001).

**Fig. 1.**
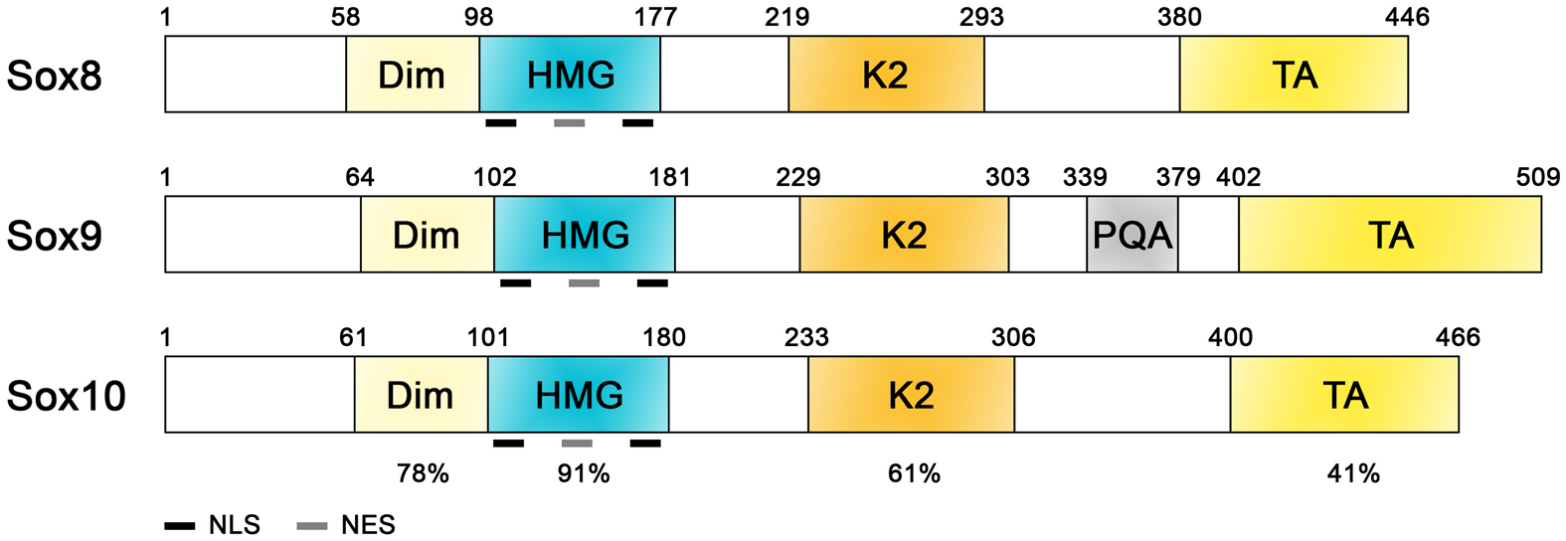
**Schematic representation of SoxE protein structure.** The TFs of the SoxE group Sox8, Sox9, and Sox10 are characterized by the presence of conserved domains such as the dimerization domain (Dim), the DNA-binding high mobility group (HMG) domain, and two distinct transactivation domains located in a central position (K2) and at the carboxy-terminal region (TA). Sox9 also contains a proline-glutamine-alanine (PQA) rich motif, absent in Sox8 and Sox10. Numbers indicate positions of amino acid residues at the beginning or end of each domain. The percentage of amino acid identity (%) between the three proteins is also shown below the conserved domains. NES, nuclear export signal; NLS, nuclear localization signal.

The Sox8 protein structure closely resembles that of its relatives, Sox9 and Sox10. Members of the SoxE group share almost 95% homology within the amino acid sequence of the HMG domain, which mediates the binding to the heptameric consensus sequence 5′-(A/T)(A/T)CAA(A/T)G-3′ (Harley et al., 1994; Schepers, 2000; Sinclair et al., 1990). The HMG domain consists of 79 amino acids arranged in three α-helices and binds to the DNA in the minor groove causing 70-85° bending toward the major groove. This bending facilitates the recruitment of other transcription factors and DNA-modifying complexes (Connor et al., 1994; Ferrari et al., 1992). In addition to the HMG domain members of the SoxE group are characterized by the presence of the dimerization domain (Dim), a central transactivation domain (named K2, also called TAM) and a transactivation domain at the carboxy-terminal of the protein (TA, also known as TAC) (Fig. 1). Following DNA-binding, transcriptional activation occurs via the cooperative action of the two transactivation regions, the K2 and TA domains (Haseeb and Lefebvre, 2019; Schepers, 2000; Schreiner et al., 2007). Sox proteins can bind to the DNA as monomers or as hetero- or homodimers (Peirano and Wegner, 2000). The Dim domain, a 38 amino acid region located before the HMG domain, plays a crucial role in facilitating dimerization. Dimerization through the Dim domain is highly context dependent, suggesting that dimerization is crucial for the regulation of specific SoxE-mediated developmental pathways. As a result of binding to the DNA as monomers or dimers, SoxE proteins activate different target genes and exert distinct effects, contributing to their functional diversity (Bridgewater et al., 2003; Schlierf et al., 2002). Recent evidence has revealed that homodimerization primarily occurs through protein interaction between the dimerization domain and the HMG, rather than between the two Dim domains (Huang et al., 2015). The importance of DNA-dependent dimerization of SoxE proteins is evident in cases where mutations impairing this process can lead to severe diseases in humans. For instance, dimerization-deficient SOX9 results in a milder form of Campomelic dysplasia without causing sex reversal (Bernard et al., 2003; Sock et al., 2003). SoxE proteins also possess two conserved nuclear localization signals (NLS) and a nuclear export signal (NES) within the HMG domain. These signals regulate the shuttling between the nucleus and cytoplasm, influencing transcriptional activity (Gasca et al., 2002; Südbeck and Scherer, 1997).

## Sox8 interplay between development and adult physiology

**Fig. 2.**
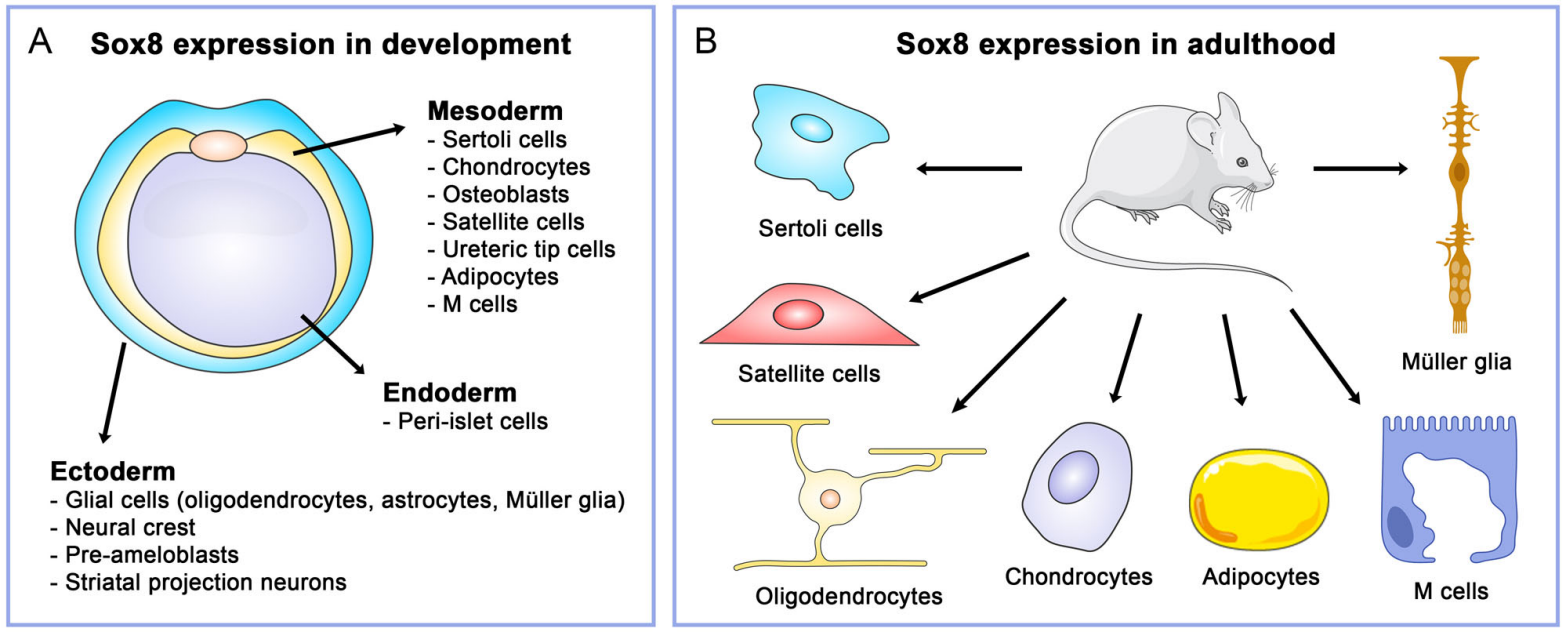
**Sox8 expression in development and adulthood.** (A) Sox8 expression in various ectoderm-, endoderm- and mesoderm-derived cell types during embryonic development. (B) Expression of Sox8 in several cell types in adult tissues.

During development, Sox8 regulates various organogenesis processes and is expressed in several ectoderm-, endoderm- and mesoderm-derived tissues. These include oligodendrocytes, astrocytes and Müller glia in the CNS, Sertoli cells in the male gonad, satellite cells in skeletal muscle, and chondrocytes and osteoblasts in the bone, among others (Fig. 2A). Sox8 is expressed in the mouse embryo already at 9.5 days *post coitum* (dpc). A detailed description of Sox8 expression during mouse and chicken development is illustrated thoroughly in Sock et al., 2001 and Bell et al., 2000, respectively. In addition to developmental regulation, Sox8 plays an important role in the maintenance of tissue homeostasis (Fig. 2B), as evidenced by the phenotype of adult *Sox8*-deficient mice, which exhibit osteopenia, reduced weight, infertility, and poor recovery in demyelinating models (Freudenstein et al., 2023; Sock et al., 2001). This section will discuss some unique functions of Sox8 from development to adulthood, while its roles in the nervous system, sex determination, and cancer will be covered in more detail in separate sections.

While Sox8 relative, Sox9*,* is often regarded as the master of chondrogenesis, various studies have provided evidence that Sox8 also contributes to chondrogenic differentiation (Chimal-Monroy et al., 2003; Herlofsen et al., 2014). Sox8 and Sox9 have been identified among the precocious markers of limb chondrogenesis with their expression preceding the induction of bone morphogenic protein (BMP) signaling. The two TFs, together with BMP signaling, orchestrate the chondrogenic program in the limb mesoderm (Chimal-Monroy et al., 2003). SOX8 expression increases during human mesenchymal stromal cell differentiation, and its absence leads to a strong reduction of *COL2A1* and several other chondrogenic genes (Herlofsen et al., 2014). Although *Sox8* knockout mice do not exhibit major skeletal malformations, they do display disturbed ossification of tarsal bones in their hindlimbs from postnatal day (P) 7 (Sock et al., 2001). This phenotype has been further characterized by Schmidt et al., who concluded that Sox8 downregulation is needed for osteoblast differentiation. Hence, its regulated expression appears to be critical for bone formation (Schmidt et al., 2005). More recently, a genome-wide association study (GWAS) identified a SNP upstream of the *SOX8* gene that is associated with human height heritability (Wood et al., 2014). Additional evidence in mice showed that Sox8 plays a significant role in skeletal growth. Molin et al. reported that Sox8 possesses independent chondrogenic capacities and can outperform Sox9 in cartilage regeneration (Molin et al., 2024). They suggested that Sox8 may be a more effective therapeutic target for enhancing chondrogenesis in skeletal malformation and degenerative conditions.

In skeletal muscle, Sox8 is highly expressed by 16.5 dpc. However, its expression decreases significantly throughout development, and by 3 months of age it is confined to satellite cells (Schmidt et al., 2003). A study by Schmidt et al. identified Sox8 as a regulator of myogenesis and proposed it as a marker of adult satellite cells. This population of cells represents an adult stem cell niche indispensable for muscle repair (Schmidt et al., 2003). A previously unreported motor phenotype in *Sox8* knockout mice has been identified by Freudenstein et al., highlighting the need for further characterization of this model to determine whether the deficit is due to skeletal, muscular, or other underlying dysfunctions. Notably, variants in the *SOX8* gene have been associated with muscular-related diseases such as familial essential tremor, severe adolescent idiopathic scoliosis, and a case of non-progressive myopathy (Clark et al., 2022; Lin et al., 2023; Warman-Chardon et al., 2023). These associations underscore the significance of understanding the role of Sox8 in skeletal muscle maintenance and regeneration.

*Sox8*-deficient mice are on average 30% lighter than wild-type littermates, yet their size is not significantly different (Sock et al., 2001). This phenotype develops independently from any muscle or bone malformation and is related to adipose tissue degeneration. These mice exhibit an increased number of Pref-1 positive cells stalling at the preadipocyte stage, with consequent depletion of the adipocyte pool and degeneration of adipose tissue (Guth et al., 2009). Moreover, these mice are more prone to weight loss in the cuprizone model of demyelination (Freudenstein et al., 2023). However, the role of Sox8 in weight loss under pathological stress in the adult has not been addressed, and it is not known if this predisposition is due to adult adipose tissue degeneration or independent unknown factors. Notably, a more recently created *Sox8*-null mouse line described by Molin et al. does not exhibit a significant weight difference in adult males compared to wild-type animals contrasting with findings from the previously studied mouse line (Molin et al., 2024). This discrepancy raises questions about the role of Sox8 in adipose tissue, highlighting the need to understand its importance in adipose tissue maintenance and development. It is important to note that the two *Sox8* knockout mouse lines were created using slightly different strategies. In the original mouse line, the *Sox8* coding sequence was replaced with a *lacZ* reporter gene, resulting in a significant reduction in body weight attributed to smaller adipose stores (Sock et al., 2001). In contrast, the newer mouse line carries only a premature stop codon and does not exhibit the weight phenotype. Molin et al. propose that the insertion of the *lacZ* gene may have disrupted the expression of a neighboring gene *Lmf1*, whose deficiency is associated with lipodystrophy (Molin et al., 2024). Nevertheless, the limited number of studies investigating Sox8's functions in adipose tissue underscores the need for further research into Sox8's role in adipose tissue development and maintenance.

A few other studies have identified a role for Sox8 in various tissues, which, to our knowledge, have not been extensively explored. Here, we provide a brief summary of this limited research. During kidney development, Sox8 together with Sox9 regulates essential pathways for kidney growth such as the RET signaling pathway. Double knockout mice for *Sox8* and *Sox9* exhibit altered ureter branching, impaired kidney formation, and hypoplasia (Reginensi et al., 2011). In the intestinal epithelium, Sox8 is present in Microfold (M) cells. These cells are part of the lymphoid tissue associated with the gut, which is responsible for antigen uptake and the initiation of the mucosal immune response. Mature M cells express the marker Gp2, which has been shown to be a direct target of Sox8. *Sox8*-deficient mice exhibit a strong reduction in the number of M cells, with consequent altered antigen uptake and germinal center activation. This suggests that Sox8 plays a crucial role in M cell differentiation, and its absence affects the antigen-specific IgA response required for proper mucosal immune reaction (Kimura et al., 2019). Sox8 expression has also been reported in the developing pancreas along with Sox10, and its presence has also been observed in the adult, in the layer of cells surrounding the pancreatic islet (Lioubinski et al., 2003). Moreover, during mouse odontogenesis, Sox8 expression is detected at the bell stage in pre-ameloblasts (Kawasaki et al., 2015). No unique roles of Sox8 have been found in these tissues to the best of our knowledge.

Despite being expressed in a variety of vital organs, and having substantial roles during development, constitutive *Sox8* knockout mice are viable, and no severe phenotype is observed in the adult. Nonetheless, further studies are required to unveil the unique functions of Sox8, as it is becoming evident that the influence of Sox8 on both developmental and adult tissue homeostasis can be of great interest for potential therapeutic regenerative implications.

## Role of Sox8 in the nervous system

During development, Sox8 is expressed in the neural crest cells which arise from the ectoderm at the border of the neural tube. This transient population of migratory stem cells gives rise to several cell lineages including melanocytes, bone, muscle, peripheral neurons, and glial cells (McCauley and Bronner-Fraser, 2006). At later stages, during neural development, Sox8 along with Sox9 is expressed in neuroepithelial precursor cells (NEPs) in the subventricular and ventricular zones of the brain and spinal cord (Pozniak et al., 2010). Expression of Sox9 from embryonic day (E) 10.5 promotes the activation of the gliogenic program in NEPs and the differentiation into oligodendrocyte precursor cells (OPCs). Sox9 expression in progenitors is followed by Sox8 and eventually Sox10, which is induced in OPCs shortly after specification and both SoxE proteins are expressed similarly throughout oligodendrocyte (OL) lineage progression and the process of maturation. *Sox9*-deficient mice show a dramatic decrease in the number of OPCs and in *Sox10*-deficient mice OL differentiation is disrupted (Hornig et al., 2013; Stolt et al., 2003). By contrast, mice lacking Sox8 expression exhibit normal OL development until birth, while at early postnatal stages, a mild phenotype is observed when OL differentiation is delayed (Stolt et al., 2004). Sox8 expression does not fully compensate for the lack of Sox9 or Sox10. In another study, in a mouse model where Sox8 replaces Sox10 expression, normal development of peripheral glial cells and sensory and sympathetic neurons is reported. However, the development of melanocytes and oligodendrocytes is impaired, and consequently, the myelination process is strongly affected. This suggests that Sox8 has a limited ability to rescue Sox10 deficiency (Kellerer et al., 2006). Additionally, the deletion of *Sox8* in *Sox9-*knockout mice enhances the Sox9-deficiency-related phenotype with the complete loss of OPC generation (Stolt et al., 2005). Reduced Sox8 expression has been suggested as a possible cause of its limited ability of phenotypic rescue in the absence of one of the other SoxE TFs (Stolt et al., 2004).

In contrast to a minor role of Sox8 during OL development, recent evidence suggests a more critical function of Sox8 in mature OLs in adult mice. Turnescu et al. reported that deletion of *Sox10* in mature OLs does not result in any myelin deficit. By contrast, additional deletion of *Sox10* in constitutive *Sox8* knockout mice causes severe myelin defects and downregulation of the myelin gene *Mog*. This study argues for a more prominent role of Sox8 in myelin maintenance and myelin gene expression (Turnescu et al., 2018). More recently, mice lacking Sox8 were challenged in the demyelinating cuprizone model. This model is characterized by heavy demyelination of the corpus callosum and allows for the study of remyelinating processes. These recent data show that the lack of Sox8 results in poor recovery. While naïve animals are fully remyelinated within 3 to 7 days post cuprizone retrieval, *Sox8*-deficient mice have significantly less myelin recovery, impaired proliferation of OPCs, and consequently a decreased number of mature OLs (Freudenstein et al., 2023).

In the vertebrate retina, glial cells known as Müller glia (MG) provide support to neurons and are essential for the maintenance of retinal homeostasis. Sox8 is a downstream target of several pathways and regulators of retinal gliogenesis, but alone Sox8 fails to promote MG development. Thus, in synergy with Notch signaling, it can activate one of the master regulators of MG differentiation, the *Lhx2* gene (de Melo et al., 2016a; McNeill et al., 2012). A shRNA approach revealed that silencing both *Sox8* and *Sox9* results in a reduction in the number of MG cells (Muto et al., 2009). However, in the absence of Lhx2, Sox8 together with Sox2 and Sox9 promotes amacrine cell specification of retinal progenitor cells at the expense of MG cells (de Melo et al., 2016b). In addition, in other brain areas, Sox8 was suggested to have a role in pituitary organogenesis by regulating the pituitary-specific transcription factor Prop1 (Nishimura et al., 2016). In the striatum, Sox8 expression is a specific marker of the embryonic direct pathway of striatal projection neurons (dSPNs), which are essential in the coordination of motor functions. Notably, *Sox8* knockout mice exhibit motor deficits in adulthood, although the cause of this deficiency has not been investigated (Freudenstein et al., 2023; Merchan-Sala et al., 2017). Sox8 is also expressed in astrocytes and OLs during cerebellar development in both mouse and chicken and more recently it has been implicated in astrocytic differentiation in the developing mouse brain (Kordes et al., 2005; Takouda et al., 2021).

Expression and functions of Sox8 during neuronal development are conserved across species. In contrast to mouse and chicken, the *Sox8* ortholog in *Xenopus* is expressed in neural crest cells before Sox9 and Sox10 and is maintained in migrating cells. In the neural plate border, Sox8 is a key regulator of the initiation of neural crest development. *Sox8* knockdown results in the delayed induction of the neural crest progenitors and consequently impaired development of several neural crest-derived lineages, suggesting a different role of SoxE proteins in this species (Bae et al., 2014; O'Donnell et al., 2006).

If not otherwise indicated, the presented data were collected from mice studies. The functions of Sox8 observed in mice and other species are likely relevant to humans, given the conserved roles of Sox family transcription factors across vertebrates. This is further supported by the association of *SOX8* mutations with several human diseases that result in neurological symptoms, as discussed more in detail in the section, ‘Implications of Sox8 in Multiple Sclerosis and other diseases’. Given these links, understanding the function of Sox8 in the murine nervous system may provide insights into its involvement in various human neurological disorders.

## Role of Sox8 in sex determination and infertility-related diseases

In mammals, sex determination occurs on a bipotent gonad that differentiates either into ovaries or testes. This decision depends on the presence of the *Sry* gene and the activation of genes required for testis differentiation, including *Sox9*, the steroidogenic factor 1 (*Sf1*) and *Dmrt1* (Koopman et al., 1991). These key male-specific determiners are critical for the activation of the anti-Müllerian hormone (Amh) and the consequent regression of the embryonic structures that give rise to the uterus (de Santa Barbara et al., 2000; Barrionuevo et al., 2016). By contrast, in the absence of the *Sry* gene, the induction of other factors such as R-spondin1 (Rspo-1) promotes the differentiation of the bipotent gonad toward ovarian development (Lavery et al., 2012).

At the time of sex determination, Sox8 collaborates with Sox9 to promote the development of male gonads (Chaboissier et al., 2004). *In vitro* studies have reported that Sox8 plays a significant role in regulating major male-determining genes including *Sf1* and *Ahm* (Schepers et al., 2003; Shen and Ingraham, 2002). Following the specification of the bipotent gonad, as well as in the postnatal testis, genetic sex reprogramming is prevented by the continuous activation of male-determining genes such as *Dmrt1*. The action of both *Sox8* and Sox9 during early embryonic development is critical for the active inhibition of ovarian differentiation (Barrionuevo et al., 2009, 2016). This is evident in mice with conditional deletion of *Sox8* and *Sox9*, which results in the downregulation of Dmrt1 and upregulation of the ovarian marker Foxl2. Consequently, this leads to the trans-differentiation of Sertoli cells into granulosa-like cells (Georg et al., 2012). Studies on sex-reversal cases have suggested that Sox8 can also independently influence sex determination. For example, in mice lacking both Sox9 and Rspo-1, Sox8 activation is sufficient to induce a pro-testis program in both XX and XY gonads (Lavery et al., 2012; Nicol and Yao, 2015; Richardson et al., 2020). Overall, these findings suggest that Sox8 plays a significant role in preventing genetic sex reprogramming and influencing sex determination independently of Sox9.

In the developing mouse testis, expression of Sox8 is detected at 12 dpc and is maintained in the adult Sertoli cells (Schepers et al., 2003). Sertoli cells produce regulatory factors of spermatogenesis and provide physical support and nutrients to the germ cells. As shown by numerous studies, deletion of *Sox8* results in adult male infertility (Guth et al., 2009; O'Bryan et al., 2008; Schepers et al., 2003; Singh et al., 2013). These mice develop an age-dependent dysfunction of the spermatogenesis process, due to the deregulation of the spermatogenic cycle and impaired integrity of the seminiferous epithelium, leading to consequent progressive male infertility (Kennedy et al., 2007; O'Bryan et al., 2008). Furthermore, the overexpression of Sox8 in a Sertoli cell line results in the upregulation of genes involved in Sertoli cell development and differentiation (Roumaud and Martin, 2019).

Sertoli cells play an essential role in spermatogenesis by establishing the blood-testis barrier (BTB), which is formed by cell–cell adhesion through tight and gap junctions. *Sox8* knockout mice show an impaired expression of several genes related to spermatogenesis and BTB establishment, including *Claudin-3*, which encodes a tight junction protein crucial for the maintenance of the BTB integrity. *In vitro* analysis also revealed that Sox8 binds directly to the promoter region of the *Claudin-3* gene and together with members of the activating protein-1 (Ap-1) family and Sf1*,* it activates the promoter of *Gja1*. This gene encodes the connexin 43 (Cx43), which is essential for cell–cell communication between Sertoli cells (Couture and Martin, 2020; Ghouili et al., 2018; Singh et al., 2013). Additionally, the lack of Sox8 leads to the disruption of microtubules, which are important for the maintenance of the seminiferous epithelium structures (Singh et al., 2013). Overall, these observations suggest that Sox8, along with other factors, provides the ideal microenvironment for the seminiferous epithelium structures by regulating the expression of structural proteins and antiapoptotic factors, thereby promoting Sertoli cell differentiation and survival (Singh et al., 2009, 2013).

Several case studies in humans have linked mutations of *SOX8* with infertility or reproductive disorders. A female patient, with XY chromosomes, displaying agonadism, skeletal and cardiac malformations, and delayed growth was found to have a higher number of CNVs in a region upstream of the *SOX8* gene (Erickson et al., 2011). Another study associated disorders of sex development (DSD) in humans with a missense mutation or chromosomal rearrangements in the region encompassing the *SOX8* gene, which result in altered protein biological function in *in vitro* assays. Additionally, this study found a significantly higher rate of *SOX8* mutations in oligospermic men and women with primary ovarian insufficiency (Portnoi et al., 2018). More recently, a novel heterozygous missense mutation in *SOX8* was identified in a 46,XY female patient with testicular regression syndrome, providing further evidence of SOX8's involvement in human reproduction (Rjiba et al., 2023).

Collectively, these findings underscore the multifaceted roles of Sox8 in both sex determination and reproductive health. Sox8, together with Sox9, is crucial for maintaining male gonadal identity and preventing genetic reprogramming toward ovarian fate. In addition, Sox8 is indispensable for Sertoli cell function and spermatogenesis, thereby playing a key role in fertility. The association of *SOX8* mutations in various human reproductive disorders further highlights its importance in both gonadal development and reproductive health.

## Implications of Sox8 in cancer

*SOX8* has been suggested to potentially function in an oncogenic manner, driving tumor development and progression. Its high expression levels are significantly correlated with poor prognosis outcomes in several cancer types, including triple-negative breast cancer (TNBC), colorectal carcinoma (CRC), endometrial carcinoma, hepatocellular carcinoma (HCC) and non-small cell lung cancer (NSCLC) (Chen et al., 2018; Dong et al., 2018; Sun et al., 2020; Tang et al., 2019; Tian et al., 2020; Wang et al., 2019; Xie et al., 2014). Oligodendrogliomas, astrocytomas, and medulloblastomas are also characterized by an increased level of SOX8 expression (Cheng et al., 2001; Schlierf et al., 2007). Additionally, SOX8 has been identified as a prognostic biomarker for glioma patients. High expression levels of SOX8 are associated with significantly lower overall survival rates and it has also been suggested to function as a predictor of therapeutic response (Jiang et al., 2020; Schlierf et al., 2007).

SOX8 appears to regulate cancer stem cell-like properties by different mechanisms. For instance, overexpression of SOX8 in TNBC cells was reported to enhance viability, migration, and proliferation and to decrease apoptosis rates (Dong et al., 2020). Accordingly, SOX8 silencing results in decreased viability and migration both in TNBC and CRC cells (Dong et al., 2020; Li et al., 2023). In HCC, contradicting studies have been published. In 2014, Zang et al. found that overexpression of SOX8 leads to increased proliferation in HCC-derived cells as well as tumor growth by promoting the activation of the Wnt/b-catenin pathway (Zhang et al., 2014). Conversely, Yang et al. reported that SOX8 limits tumor growth by promoting ferroptosis (Yang et al., 2023). SOX8 has been identified as a possible target for treating chemotherapy-resistant cancers. In cisplatin chemo-resistant tongue squamous cell carcinoma (TSCC), SOX8 is responsible for the induction of stem cell-like and chemoresistance properties through the activation of the Wnt/b-catenin pathway (Xie et al., 2018). Following *SOX8* knockdown, TSCC cells become responsive to cisplatin treatment and lose their ability to form tumor spheres. A recent study also associated SOX8 with chemoresistance in ovarian cancer. Similar observations were found in CRC where SOX8 was shown to be involved in promoting resistance to cetuximab, an epidermal growth factor receptor inhibitor used for treatment against metastatic CRC, head, and neck cancer (Piao et al., 2022; Sun et al., 2020). Additionally, in castration-resistant prostate cancer, *SOX8* knockdown results in a reversal of enzalutamide resistance (Du et al., 2022). Overall, evidence suggests that SOX8 could be a promising target for treating drug-resistant cancers, as it appears to play a significant role in promoting chemoresistance. However, the limited number of studies on SOX8 in cancer underscores the need to broaden our understanding of its potential as an oncogene with therapeutic interest.

## Implications of Sox8 in Multiple Sclerosis and other diseases

**Fig. 3.**
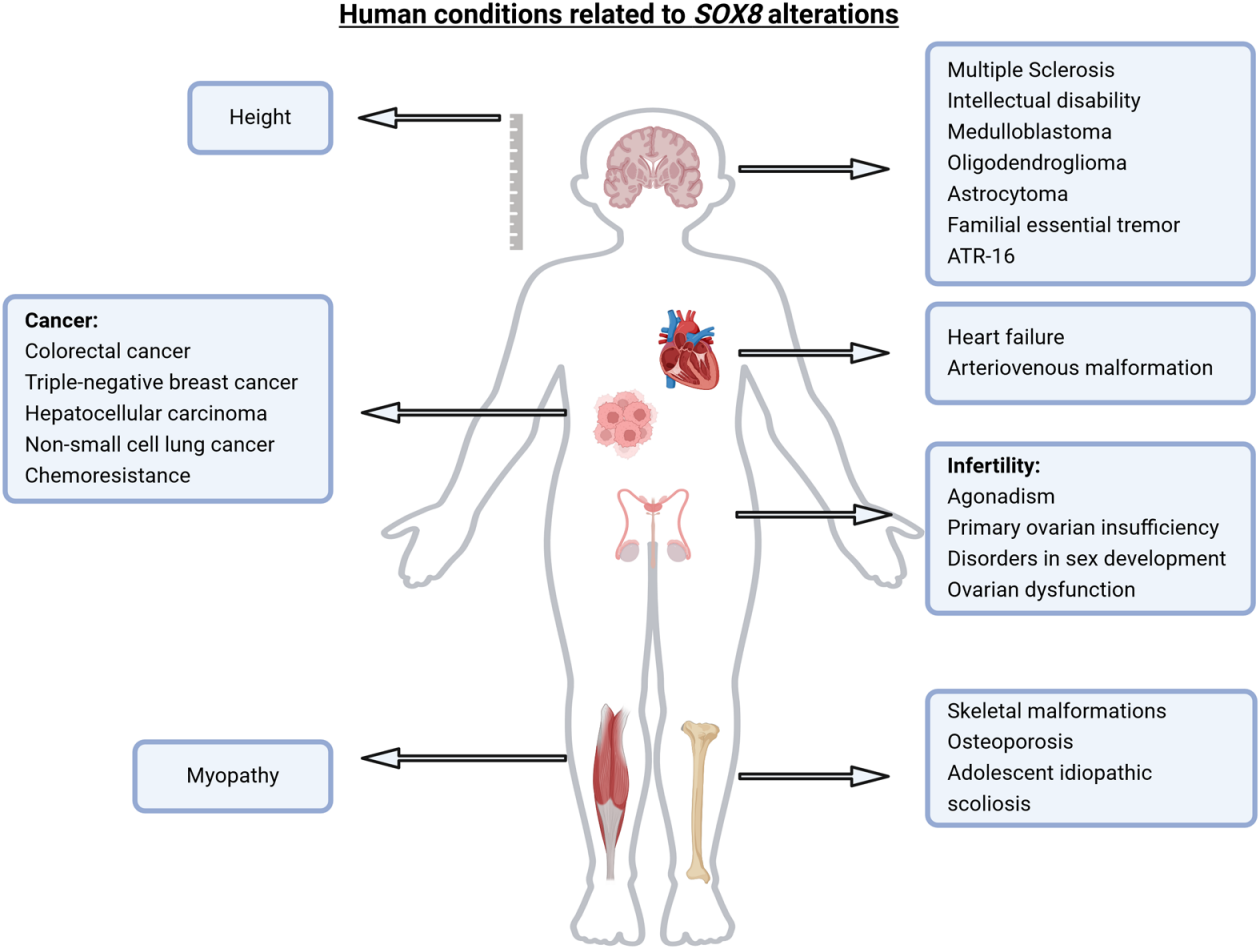
**SOX8 implications in human diseases.** Schematic representation summarizing the diverse implications of SOX8 across different human pathological conditions. For detailed studies and diseases, refer to Table 1. Figure created with BioRender.com.

In recent years, several studies have correlated anomalies of the *SOX8* gene with a variety of diseases (Fig. 3; Table 1). For instance, two large GWAS analyzed the genetic loci that are associated with a higher risk of developing multiple sclerosis (MS). Among the potential risk factors identified, a recurrent SNP rs2744148, located 36 kb downstream of the *SOX8* gene, was found in patients affected by MS (Lill et al., 2013; Sawcer et al., 2011). However, the role of this SNP and its association with MS remains unclear. Further research is required to investigate whether it influences *SOX8* expression, potentially affecting either the transcriptional regulation of the gene or the resulting protein product. Interestingly, while Sox8 appears to be dispensable during developmental myelination, recent data suggest that this TF is critical for myelin maintenance and myelin gene expression in the adult mouse brain (Turnescu et al., 2018). More recently, a study on a demyelinating model of the mouse brain demonstrated that Sox8 is necessary for timely remyelination in the CNS. The study reported an impaired proliferation of OPCs in *Sox8*-deficient mice that results in an inadequate pool of mature OLs, which in turn impairs the remyelination process (Freudenstein et al., 2023). The identification of *SOX8* as a gene of interest in MS highlights the need to better understand its role in remyelinating processes and myelin maintenance in the adult brain.

**Table 1.** Summary of the current studies on SOX8 implication in human disorders

Reference	Associated disease	SOX8 implication
Chen et al., 2018 Dong et al., 2018 Tang et al., 2019	Triple-negative breast cancer	Upregulation of SOX8 negatively correlates with prognosis outcomes Maintenance of stem-like capacities
Wang et al., 2019 Li et al., 2023 Piao et al., 2022	Colorectal cancer	High expression of SOX8 negatively correlates with prognosis outcomes Cetuximab resistance
Zhang et al., 2014 Yang et al., 2023	Hepatocellular carcinoma *Contradicting results	SOX8 overexpression increases HCC cell proliferation leading to tumor growth SOX8 limits tumor growth via ferroptosis
Sun et al., 2020	Ovarian Cancer	Chemoresistance (cisplatin)
Tian et al., 2020	Endometrial carcinoma	Higher expression of SOX8 is linked to poor prognosis
Xie et al., 2014	Non-small cell lung cancer	Higher expression of SOX8 is linked to poor survival rates
Schlierf et al., 2007 Cheng et al., 2001 Jiang et al., 2020	Oligodendrogliomas Astrocytomas Astrocytomas, Medulloblastomas Gliomas	Enhanced expression of SOX8 is related to lower survival rates
Xie et al., 2018	Tongue squamous cell carcinoma	Chemoresistance (cisplatin)
Du et al., 2022	Castration-resistant prostate cancer	Enzalutamide resistance
Sawcer et al., 2011 Lill et al., 2013	Multiple sclerosis	*SOX8* SNP rs744148 is associated with a higher risk of developing multiple sclerosis
Clark et al., 2022 Lin et al., 2023 Warman-Chardon et al., 2023 Liu et al., 2022	Familial essential tremor Adolescent idiopathic scoliosis Non-progressive myopathy Heart failure	Genetic variant of *SOX8* associated with disease
Wood et al., 2014	Height	Genetic variant of *SOX8* associated with height heritability
Pfeifer et al., 2000 Gibson et al., 2008	ATR-16	*SOX8* suspected as a disease-causing gene
Sasahara et al., 2007 Liu et al., 2019	Arteriovenous malformation Osteoporosis	*SOX8* is differentially expressed in microarray data analysis
Erickson et al., 2011 Portnoi et al., 2018 Rjiba et al., 2023	Infertility and disorders of sex development	*SOX8* higher CNV in female patients with agonadism and male XY chromosomes *SOX8* missense mutation or chromosomal rearrangements are linked to oligospermic men and primary ovarian insufficiency in women *SOX8* heterozygous missense mutation identified in female patients with testicular regression syndrome

Additionally, Freudenstein et al. revealed a previously unreported motor deficit in naïve *Sox8* knockout mice (Freudenstein et al., 2023). The underlying cause of this motor deficit is currently unknown. Considering the widespread expression of Sox8 across various tissues, it is possible that this deficit might be related to muscular, skeletal, or neurological factors. Further studies are necessary to thoroughly characterize the phenotype of this mouse line. Interestingly, genetic studies have associated variants in the *SOX8* gene with familial essential tremor, severe adolescent idiopathic scoliosis, and heart failure (Clark et al., 2022; Lin et al., 2023; Liu et al., 2022). Moreover, biallelic *SOX8* variants were identified in a case study of a patient displaying non-progressive myopathy, skeletal dysplasia, intellectual delay, and ovarian dysfunction (Warman-Chardon et al., 2023).

*SOX8* has been proposed as a candidate gene that contributes to the phenotype of ATR-16 patients. ATR-16 is a disease caused by a terminal deletion in the 16p13.3 chromosome, resulting in haploinsufficiency of multiple genes and characterized by intellectual disability, α-thalassemia, and dysmorphic features. In patients affected by ATR-16, heterozygous deletion in the region encompassing the *SOX8* gene is sufficient to induce the phenotype of ATR-16, suggesting a possible role of SOX8 in this disease (Gibson et al., 2008; Pfeifer et al., 2000). However, due to the rarity of the disease, no definitive link between *SOX8* deletion and ATR-16 has been proven and a combined haploinsufficiency may also be a cause for the disease. Other diseases involving altered *SOX8* gene expression include brain arteriovenous malformation and osteoporosis. Microarray data analysis identified *SOX8* among the differentially expressed genes in both disorders (Liu et al., 2019; Sasahara et al., 2007).

*Sox10* heterozygous mice exhibit aganglionosis of the colon and pigmentation defects, making them a suitable model for human Waardenburg–Hirschsprung disease (Herbarth et al., 1998). While Sox8 alone does not seem to influence the enteric nervous system development, the additional deletion of *Sox8* in *Sox10-*heterozygous mice leads to increased apoptosis in progenitors of the vagal neural crest. This results in more pronounced defects in the enteric nervous system development compared to *Sox10* heterozygous mice alone, suggesting that both Sox8 and Sox10 are essential for the maintenance of these vagal neural crest stem cells (Maka et al., 2005). In summary, *SOX8* has emerged as a gene of interest in several pathologies, often associated with infertility, muscle, skeletal, and neurological deficits. Further research on the role of SOX8 in diseases will be crucial to uncover its potential as a therapeutic target.

## Conclusions and future directions

The TF Sox8 shares many similarities in the molecular structure and expression pattern with its relatives Sox9 and Sox10. Together, SoxE proteins function in synergy, modulating, compensating, and regulating each other. However, while the functions of Sox9 and Sox10 are better understood and mutations in these two genes lead to striking phenotypic effects, the precise role of Sox8 remains somewhat elusive. Nevertheless, recent studies have revealed its essential regulatory role in adult tissue homeostasis, highlighting its potential as an interesting therapeutic target.

Altogether, despite its limited importance as a developmental regulator, Sox8 has emerged as a crucial modulator of adult tissue homeostasis and the maintenance of adult stem cell pools. In muscle, Sox8 plays a minor role during development, yet in the adult, it is expressed in satellite muscle cells, which are essential for muscle regeneration. Sox8 also exhibits more effective chondrogenic capacities compared to Sox9, making it an interesting candidate for cartilage regeneration. Moreover, the lack of Sox8 results in a slower recovery in the demyelinating model of cuprizone, indicating that Sox8 alone may be relevant for OPC response in demyelinating lesions. In adipose tissue, *Sox8* knockout mice exhibit degeneration in adulthood. However, whether this degeneration is due to the inability to replace cells from an existing precursor pool remains unclear. Additionally, impaired Sox8 function in the maintenance of Sertoli cells and the BTB causes severe dysregulation of spermatogenesis with consequent male infertility. Altogether, the evidence that Sox8 is required for tissue homeostasis and maintenance points to its potential implication in diseases. Notably, *SOX8* has been identified as a gene of interest in a variety of disorders and its regulatory roles extend from cancer stem cell properties to chemoresistance. A notable limitation that has emerged from studies on constitutive *Sox8* knockout mice is the incomplete characterization of the available mouse lines, leaving many aspects unexplored. Further characterization of this mouse model could provide valuable insights into the role of Sox8 in adult tissue physiology, particularly in the context of disease and injury models.

In conclusion, while the specific role of Sox8 is not fully understood, emerging evidence suggests that this TF is an essential contributor to various biological processes, homeostasis of adult tissues, and regeneration. Sox8 might be a promising potential therapeutic candidate, particularly for regenerative and cancer treatments. This review highlights the lack of knowledge in understanding Sox8 functions and emphasizes the need for further investigation. Future studies aimed at elucidating the molecular mechanisms underlying Sox8's roles in physiological and pathological conditions will pave the way for innovative therapeutic strategies and clinical interventions.
